# SARS‐CoV‐2 omicron variant may present with severe sickle cell painful crisis: A report of two cases

**DOI:** 10.1002/ccr3.5934

**Published:** 2022-06-09

**Authors:** Elrazi Ali, Ahmed Hatim, Mohamed Yassin

**Affiliations:** ^1^ 36977 Internal Medicine Department Hamad Medical Corporation Doha Qatar; ^2^ 36977 Department of Hematology and Medical Oncology Hamad Medical Corporation Doha Qatar

**Keywords:** COVID‐19, omicron, SARS‐CoV‐2, sickle cell anemia, sickle cell disease

## Abstract

Coronavirus disease 2019 (COVID‐19) is a respiratory viral illness that is caused by coronavirus 2 (SARS‐CoV‐2). The disease often presents with non‐specific symptoms such as fever, headache, and fatigue, accompanied by respiratory symptoms (e.g., cough and dyspnea) and other systemic involvement. Currently, the virus had shown significant changes and mutations that resulted in the emergence of different strains. Each strain varies in its virulence, disease severity, and the response of the body's immune system. Sickle cell disease characterized by hemolytic anemia particularly in associated with stress. Patients with sickle cell disease infected with SARS‐CoV‐2 are reported to have increased risk for hospitalization, thrombosis, and other complications compared with non‐sickle cell patients. The Omicron variant causes mild disease in general population; however, in patients with sickle cell disease, the data are limited. We present two patients known to have sickle cell disease presented with a severe acute painful crisis that required hospitalization after infection with Omicron variant of the SARS‐CoV‐2 virus.

## Graphical abstract

SARS‐CoV‐2 Omicron variant causes mild COVID‐19 infection in most patients; however, it may result in hospitalization in patients with sickle cell anemia due to severe painful crisis.

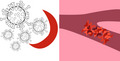

## 1 BACKGROUND

Severe acute respiratory syndrome coronavirus 2 (SARS‐CoV‐2) is a novel coronavirus, was identified as the source of a cluster of pneumonia cases in Wuhan, Hubei Province, China, near the end of 2019. It quickly spread over the world, resulting in a global epidemic. The disease severity is more pronounced in patients with comorbidities.^1^, ^2^ The presentation of SARS‐CoV‐2 infection is mainly with respiratory symptoms, but other major organ involvement liver, kidney, pancreas, and skin are also seen.^3^, ^4^, ^5^ Despite the availability of protective vaccines, SARS‐CoV‐2 continued to develop mutations in the spike proteins which resulted in the emergence of new stains. Until now, there is Alfa, Beta, Gamma, Delta, and lastly, Omicron variants. Sickle cell anemia is a hematological disorder with sickled hemoglobin, which is less soluble. This leads to increased red blood cells sickling and destruction. The most common presentation of sickle cell disease is vaso‐occlusive crisis. The severity of the vaso‐occlusive crisis varies depending on the genotype of sickle cell disease; it is more severe with genotype SS and less severe with HbSE.^6^, ^7^ Generally, the vaso‐occlusive crisis is managed with hydration, analgesics, and oxygen; if patients did not improve, they might require blood transfusion.^8^ Among the common trigger of acute painful crisis is infections. Little is known about the effect of the Omicron statin of SARS‐CoV‐2 virus on patients with sickle cell disease. We are reporting two cases of sickle cell disease who developed a severe painful crisis after infection with the Omicron variant of the SARS‐CoV‐2 virus.

## 2 CASE PRESENTATION

### 2.1 Case 1

A 39‐year‐old women with sickle cell disease presented to the emergency department with a complaint of pain. She has a past medical history of sickle cell disease with recurrent vaso‐occlusive crisis on criznalizumab and hydroxyurea 1000 mg twice daily and past surgical history of cholecystectomy, tonsillectomy, and hip replacement. She was vaccinated with SARS‐CoV‐2 (Pfizer) and received 2 doses, first dose was on May 05, 2021, and the second on May 26, 2021, and it was 8 months prior to this admission. She presented with generalized body pain, fever, and dry cough for 2 days. The pain was severe 10/10 in severity, associated with severe headache but no shortness of breathing and no other systemic manifestations. The condition started for 1 day and was not responding to NSAID and paracetamol. Vital signs were within normal range, and physical examination was unremarkable. Laboratory investigations showed Hg 7.1, and patient was transfused 1 unit of packed RBC. Other laboratories demonstrated high reticulocytes count, normal creatinine, and other investigations in Table 2. Chest X‐ray was unremarkable apart from prominent bronchovascular and linear atelectatic band at the left lung base (Figure 2A). Because of the respiratory symptoms, respiratory viral panel was sent and she tested positive for SARS‐CoV‐2 Omicron type with ct value of 29.21. She did not require oxygen and diagnosed with mild illness.^9^ She was admitted for pain management was initially started on celecoxib and paracetamol but the patient was still experiencing pain and morphine 5 mg subcutaneous every 6 h was added. Her pain then improved with IV hydration and analgesia and after being controlled, she was switched back to paracetamol and celecoxib. She stayed in the hospital for 9 days after being tested negative and discharged home.

### 2.2 Case 2

A 52‐year‐old Nigerian female is known to have diabetes mellitus and sickle cell disease (HbSS Variant). She presented to the emergency department in December 2021 with complaints of central chest pain, left‐sided hip pain, and on and off back pain for 2 days. Her chest pain was mild to moderate, not associated with exertion, aching in nature, not associated with palpitations, no shortness of breath, no orthopnea, no paroxysmal nocturnal dyspnea, no lower limb swelling, and no loss of consciousness. The pain occurred subsequently with her hip pain and back pain. The systemic review was unremarkable for fever, weight loss, and no constitutional symptoms. She was vaccinated and received two doses of SARS‐CoV‐2 (Moderna Vaccination). First dose being on April 06, 2021, and the second dose on May 08, 2021. The latter being almost 7 month prior to admission. Her home medications include metformin 500 mg twice daily and folic acid 5 mg daily, and she was not on hydroxyurea. Patient had history of 3 sickle painful crisis with admission in the hospital in 2017, 2018, and 2019, respectively. She was following regularly with the hematology team.

**FIGURE 1:**
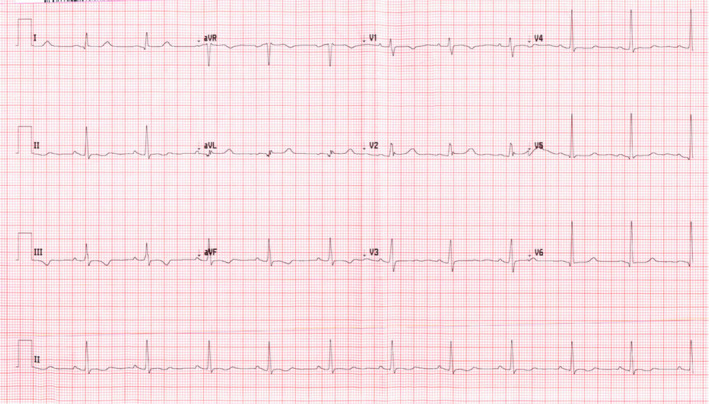
shows the ECG of case 2 with sinus rhythm with t wave abnormalities

**FIGURE 2:**
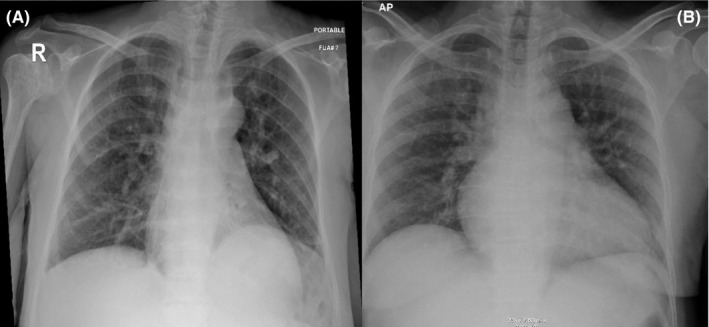
(A) Poterioanterior chest x‐ray of patient 1 showing prominent bronchovascular markings in the lungs bilaterally with linear atelectatic band at the left lung base with no consolidation. (B) Poterioanterior chest x‐ray of patient 2 showing prominent bronchovascular markings, alveolar‐interstitial pattern with no obvious consolidation

Upon initial assessment, her blood pressure was 136/80 mmHg, oxygen saturation was 98% on ambient air, pulse rate was 80 beats per minute, and respiratory rate of 18 breaths per minute. Her body weight was 64 kg and BMI 21.97. Physical examination was unremarkable apart from pallor. Initial laboratories were significant for a total leukocyte count of 13.5 × 103 Ul with peripheral neutrophilia of 7.2 × 103 Ul. Hemoglobin was 7.7 g/Dl with a reticulocyte count of 185 × 103 Ul and a reticulocyte percentage of 7.2%. Other metabolic panels, including liver and renal function, were within normal limits (Tables 1 and 2). ECG showed normal sinus rhythm with old changes (Figure 1). Because he had some mild dry cough and as part of precautions of the ongoing COVID‐19 pandemic, COVID‐19 Antigen rapid test was ordered, which came back positive. Subsequently, nasopharyngeal/throat swab was tested using fully automated reverse‐transcription polymerase chain reaction (RT‐PCR) Cobas^®^ 6800 (Roche), and the result was positive for SARS‐CoV‐2 with a ct value of 18.75. A chest X‐ray was done, which elucidated diffuse reticular‐like opacities of the lungs more accentuated at the bases, compatible with alveolar‐interstitial pattern, and the impression was COVID pneumonia (Figure 2B). The patient was transferred to a COVID facility and diagnosed as a case of a painful crisis with mild COVID pneumonia (mild illness).^9^ The patient was started on favipiravir. During her hospital stay, patient had ongoing pain all around her body and as per the patent it was similar to the one she had during her previous visits to the hospital. Patient required tramadol oral 50 mg BID for 2 days which was then switched to Morphine 5 mg subcutaneous every 6 h along with regular paracetamol and diclofenac. These medication were then switched to PRN medication as she showed some improvement and her pain got better. During her hospital stay, patient did not require oxygen and there was no evidence of any organ damage. Patient did not require any blood transfusion as her hemoglobin levels were plateauing around 7.5–8. She was kept in isolation for 7 days for observation and then was discharged home.

**TABLE 1:** Blood investigation for case 1

	Result	Reference		Result	Reference
WBC	13.5 × 10^3^/μl	4.0–10.0	Absolute neutrophil count #	7.2 × 10^3^/μl	2.0–7.0
RBC	2.6 × 10^6^/μl	4.5–5.5	Lymphocyte #	5.0 × 10^3^/μl	1.0–3.0
Hemoglobin	7.7 gm/dl	13.0–17.0	Monocyte #	0.7 × 10^3^/μl	0.20–1.0
Hct	22.9%	40.0–50.0	Eosinophil #	0.4 × 10^3^/μl	0.0–0.5
MCV	88.0 fl	83.0–101.0	Basophil #	0.20 × 10^3^/μl	0.02–0.10
MCH	29.7 pg	27.0–32.0	Neutrophil percentage	53.7%	
MCHC	33.8 gm/dl	31.5–34.5	Lymphocyte percentage	37.0%	
RDW‐CV	17.1%	11.6–14.5	Monocyte percentage	5.3%	
Platelet	236 × 10^3^/μl	150–400	Eosinophil percentage	2.7%	
MPV	9.6 fl	7.4–10.4	Basophil percentage	1.3%	
Urea	5.6 mmol/L	3.20–7.40	Bilirubin T	17.9 μmol/L	3.40–20.50
Creatinine	50 μmol/L	63.60–110.50	Total protein	67 gm/L	64.0–83.0
Sodium	134 mmol/L	135.0–145.0	Albumin	38 gm/L	35.0–50.0
Potassium	4.0 mmol/L	3.60–5.10	ALP	51 U/L	40.0–150.0
Chloride	105.6 mmol/L	96.0–110.0	ALT	24 U/L	0.0–55.0
Bicarbonate	22.8 mmol/L	22.2–29.0	AST	26 U/L	5.0–34.0
Adjusted calcium	2.20 mmol/L				

**TABLE 2:** Blood investigation for patient 2

	Result	Reference		Result	Reference
COVID‐19 PCR	Positive		Creatinine	44 μmol/L	44–80
COVID‐19 Average CT	29.21		Sodium	141 mmol/L	133–146
WBC	13.7 × 10^3^/μl	4.0–10.0	Potassium	4.4 mmol/L	3.5–5.3
Hgb	7.2 gm/dl	12.0–15.0	Chloride	110 mmol/L	95–108
Platelet	361 × 10^3^/μl	150–400	Bicarbonate	20 mmol/L	22–29
MPV	10.8 fl	7.4–10.4	Calcium	2.10 mmol/L	
Absolute Neutrophil count Auto# (ANC)	7.6 × 10^3^/μl	2.0–7.0	Adjusted calcium	2.28 mmol/L	2.20–2.60
Eosinophil Auto #	0.2 × 10^3^/μl	0.0–0.5	Bilirubin T	61 μmol/L	0–21
Basophil Auto #	0.11 × 10^3^/μl	0.02–0.10	Bilirubin D	27 μmol/L	0–5
Urea	1.9 mmol/L	2.5–7.8	Total protein	68 gm/L	60–80
CRP	3.9 mg/L	0.0–5.0	Albumin Lvl	31 gm/L	35–50
Retic #	669.4 × 10^3^/μl	50.0–100.0	Alk Phos	134 U/L	35–104
Retic %	>24.5%	0.5–2.5	ALT	83 U/L	0–33
Ferritin	9823 mcg/L	8–252	AST	103 U/L	0–32

## 3 DISCUSSION

Sickle vaso‐occlusive crisis can be precipitated by various stressors; these include fever, infection, cold, exercise,^10^ and occasionally, no trigger is identified. One of the spreading viral infections is SARS COV‐2. Recently, many variants of the SARS‐COV2 virus have emerged. The new variants resulted in pandemic waves of the COVID‐19, to which people are less protected by vaccination compared with the wild virus (alpha variant). Omicron (B.1.1.529 lineage) was first reported from Botswana and soon thereafter from South Africa in November 2021. The emergence of the Omicron variant resulted in a huge pandemic wave that involved millions of people. Omicron is characterized by a high replication rate compared with other stains,^11^ ability to escape the humoral immune response, high rate of reinfection,^12^ and lower severity and hospitalization.^13^ Most of the reports showed that Omicron causes milder disease. However, the effect on patients with sickle cell anemia is not well known.

The data regarding COVID‐19 infection on sickle cell patients is scarce, mainly on the wild (alpha) variant. Data showed that patients with sickle cell disease reported having an increased risk of the development of thrombotic complications and acute chest syndrome.^14^, ^15^ With SARS‐CoV‐2, infection, the differentiation between the acute chest syndrome and COVID‐19 pneumonia is complicated, as COVID‐19 pneumonia can have radiological changes in chest X‐ray similar to acute chest syndrome. In addition, the inflammatory response resulting from the COVID‐19 infection can precipitate acute chest syndrome. As a result, physicians need to treat acute chest syndrome, which usually responds to blood exchange.^16^ With the Omicron variant, our reported cases showed that sickle cell patients presented with painful crisis and mild COVID‐19 illness. The two patients developed a mild COVID‐19 infection, but the course of the illness was complicated by a severe painful crisis that required hospitalization. This means that Omicron SARS‐CoV‐2 infection may end in hospitalization by precipitating a severe painful crisis in patients with sickle cell anemia despite the lesser severity of respiratory symptoms.

Moreover, the Omicron strain makes the effect of the infection in sickle cell patients more complex as currently, most of the population, including patients with sickle cell disease, have received vaccinations. However, the effect on patients who were not immunized or who had not encountered the virus before is not clear. It is not known if unvaccinated patients with sickle cell anemia will develop severe respiratory symptoms, despite the fact that previous immunization has little effect on Omicron infection.

## 4 CONCLUSION

The Omicron variant of SARS COV‐2 may present with severe sickle cell crisis in patients with sickle cell anemia who were vaccinated resulting in hospitalization. This is in contrast to the general population, where Omicron is usually milder and requires no hospital admission. However, a large‐scale study is needed for a better understanding of the Omicron effect in patients with sickle cell anemia.

## AUTHOR CONTRIBUTIONS

Elrazi Ali, Ahmed Hatem, and Mohamed Yassin involved in writing, editing, and final approval of the manuscript.

## CONFLICT OF INTEREST

All authors have no conflict of interest.

## ETHICAL APPROVAL

The case was approved by medical research center with MRC‐04–22–057.

## CONSENT

Written informed consent was obtained from the patient to publish this report in accordance with the journal's patient consent policy.

## Data Availability

Data are available on reasonable request.
